# A rare *CHD5* haplotype and its interactions with environmental factors predicting hepatocellular carcinoma risk

**DOI:** 10.1186/s12885-018-4551-y

**Published:** 2018-06-15

**Authors:** Qin Xiao, Lianzhou Chen, Haiqing Luo, Hongmei Li, Qingming Kong, Fei Jiao, Shifeng Pang, Ming Zhang, Feifei Lan, Wenguo Fan, Hui Luo, Tao Tao, Xiao Zhu

**Affiliations:** 1 Guangdong Provincial Key Laboratory of Medical Molecular Diagnostics, Dongguan Scientific Research Center, Guangdong Medical University, Dongguan, China; 2 Department of Blood Transfusion, Peking University Shenzhen Hospital, Shenzhen, China; 3 Digestive System Tumor Tissue Bank, Center of Surgery Laboratory, The First Affiliated Hospital, Sun Yat-sen University, Guangzhou, China; 4 The Affiliated Hospital Cancer Center, Guangdong Medical University, Zhanjiang, China; 5 Department of Pathology, Guangdong Medical University, Dongguan, China; 6 Immunity and Biochemical Research Lab, Zhejiang Academy of Medical Sciences, Hangzhou, China; 7 Department of Biochemistry and Molecular Biology, Binzhou Medical University, Yantai, China; 8 Department of Gastroenterology, Zibo Central Hospital, Zibo, China; 9 Forensic Identification Institute, Guangdong Women and Children Hospital, Guangzhou, China; 10 Guanghua School of Stomatology, Hospital of Stomatology, Sun Yat-sen University, Guangzhou, China

**Keywords:** CHD5, Gene haplotype, Hepatocellular carcinoma, Alcohol intake, Risk

## Abstract

**Background:**

*CHD5* is a conventional tumour-suppressing gene in many tumours. The aim of this study was to determine whether *CHD5* variants contribute to the risk of hepatocellular carcinoma (HCC).

**Methods:**

Gene variants were identified using next-generation sequencing targeted on referenced mutations followed by TaqMan genotyping in two case-control studies.

**Results:**

We discovered a rare variant (haplotype AG) in *CHD5* (rs12564469-rs9434711) that was markedly associated with the risk of HCC in a Chinese population. A logistical regression model and permutation test confirmed the association. Indeed, the association quality increased in a gene dose-dependent manner as the number of samples increased. In the stratified analysis, this haplotype risk effect was statistically significant in a subgroup of alcohol drinkers. The false-positive report probability and multifactor dimensionality reduction further supported the finding.

**Conclusions:**

Our results suggest that the rare *CHD5* gene haplotype and alcohol intake contribute to the risk of HCC. Our findings can be valuable to researchers of cancer precision medicine looking to improve diagnosis and treatment of HCC.

**Electronic supplementary material:**

The online version of this article (10.1186/s12885-018-4551-y) contains supplementary material, which is available to authorized users.

## Background

Hepatocellular carcinoma (HCC) is the most common primary liver cancer and has the worst prognoses of all malignancies. The etiological background of HCC patients differs between patients from different regions. In China, chronic hepatitis B virus (HBV) infection is the most important risk factor for HCC; two-thirds of the worldwide HBV carriers are Chinese, and approximately 20% of them have a chronic HBV infection [1].

*Chromodomain helicase DNA-binding protein 5* (*CHD5*) is on the *Homo sapiens* chromosome 1p36.31. It is one of the nine members of the CHD-binding enzymes and belongs to the snf2 DNA helicase/methylase superfamily [2]. *CHD5* consists of 42 exons coding for a 223 kDa protein. Based on its protein sequence, it contains two PHD zinc fingers, two chromodomains and a helicase/ATPase domain.

Evidence that *CHD5* functions as a tumour suppressor in human cancers has emerged principally from studies of neuroblastoma, wherein loss of the CHD5 locus on chromosome 1p36.3 is very common. *CHD5* has garnered considerable interest owing to its ability to severely impact clonogenicity and tumourigenecity. Although its expression was thought to be restricted to neural-related tissues, it was subsequently found to be a tumour suppressor in neuroblastoma [3], melanoma [4], lung cancer [5], breast cancer [6], ovarian cancer [7], gastric cancer [8], colorectal cancer [9] and HCC [10]. CHD5 loss leads to a wide range of cellular consequences, and it, therefore, remains a promising candidate for further investigation in HCC. In this study, we tested the hypothesis that single-nucleotide polymorphisms (SNPs) in the 1p36 region of *CHD5* are associated with HCC.

## Methods

### Study subjects

First, 280 unrelated HCC patients and 255 healthy controls (admitted to the Zibo Central Hospital in North China between 2006 and 2010) were recruited for our study. Then, 549 HCC patients and 510 controls (admitted to the Peking University Shenzhen Hospital between 2007 and 2010, the First Affiliated Hospital at the Sun Yat-Sen University between 2007 and 2015, and the Cancer Hospital of Guangzhou Medical University between 2009 and 2011 in South China) were enrolled in the replication study. The selection criteria for the controls included no individual/family history of cancer or diabetes; no history of HBV, HCV, tuberculosis or HIV infection and frequency of age (± 5 years) and sex matching those of the patients. All patients were newly diagnosed, previously untreated (no radiotherapy or chemotherapy) and were proven to have no other tumours. We used published diagnostic criteria for HCC [11, 12]. The definition of ‘Ever or current smokers’ is those who had smoked more than 100 cigarettes, which is equal to five packs in their whole life before the date they were diagnosed with cancer or before the date they were interviewed for the controls [13, 14]. The definition of ‘Ever or current drinkers’ were those who have consumed alcoholic beverages ≥one time per week for 6 months or more previously; otherwise, they were defined as non-drinkers [15]. The purpose of frequency matching was to control confounding factors while evaluating the main effect of *CHD5* polymorphisms. All patients and controls were *Han* Chinese in origin and lived in China. Relevant biographical features of the subjects are summarised in Table 1.

**Table 1:** Clinical and laboratory features of the subjects included in the study

Characteristics	Discovery study			Replication study			Combined study		
	Cases (%)	Controls (%)	*P*	Cases (%)	Controls (%)	*P*	Cases (%)	Controls (%)	*P*
*n*	280	255		549	510		829	765	
Age (ys, mean ± SD)	55.1 ± 14.6	41.5 ± 9.1	< 0.001^a^	56.6 ± 11.3	47.2 ± 10.7	< 0.001^a^	56.0 ± 13.6	44.8 ± 10.3	< 0.001^a^
Gender (F/M)	53/227	91/164	< 0.001^b^	125/424	167/343	< 0.001^b^	178/651	258/507	< 0.001^b^
Smoking	99 (35.36)	56 (21.96)	0.001b	231 (42.08)	145 (28.43)	< 0.001^b^	330 (39.81)	201 (26.27)	< 0.001^b^
Missing	5 (1.79)	7 (2.75)		22 (4.01)	26 (5.10)		27 (3.26)	33 (3.98)	
Drinking	95 (33.93)	54 (21.18)	0.001^b^	210 (38.25)	129 (25.29)	< 0.001^b^	305 (36.79)	183 (23.92)	< 0.001b
Missing	8 (2.86)	7 (2.75)		28 (5.10)	29 (5.69)		36 (4.34)	36 (4.71)	
HBsAg+	224 (80.00)	0 (0.00)		419 (76.32)	0 (0.00)		643 (77.56)		
Anti-HCV	0 (0.00)	0 (0.00)		4 (0.73)	0 (0.00)		4 (0.48)	0 (0.00)	
Anti-HIV	0 (0.00)	0 (0.00)		2 (0.36)	0 (0.00)		2 (0.24)	0 (0.00)	
Serum AFP (>25 μg/L)	233 (83.21)	0 (0.00)		431 (78.51)	0 (0.00)		664 (80.10)	0 (0.00)	
Tumor size (cm)									
≤5	65 (23.21)			139 (25.32)			204 (24.61)		
>5, ≤10	93 (33.21)			273 (49.73)			366 (44.15)		
>10	122 (43.57)			137 (24.95)			259 (31.24)		
Cirrhosis									
No	16 (5.71)			38 (6.92)			54 (6.51)		
Yes	260 (92.86)			504 (91.80)			764 (92.16)		
Missing	4 (1.43)			7 (1.28)			11 (1.33)		
Tumor morphology									
No residual tumor	19 (6.79)			43 (7.83)			62 (7.48)		
Uninodular tumor	55 (19.64)			89 (16.21)			144 (17.37)		
Multinodular tumor	107 (38.21)			228 (41.53)			335 (40.41)		
Massive tumor	92 (32.86)			168 (30.60)			260 (31.36)		
Missing	7 (2.50)			21 (3.83)			28 (3.38)		
Differentiation									
Well	31 (11.07)			77 (14.03)			108 (13.03)		
Moderate	78 (27.86)			195 (35.52)			273 (32.93)		
Poor	171 (61.07)			277 (50.46)			448 (54.04)		
Metastasis									
Abscent	81 (28.93)			189 (34.43)			270 (32.57)		
Present	193 (68.93)			347 (63.21)			540 (65.14)		
Missing	6 (2.14)			13 (2.37)			19 (2.29)		
TNM stage									
I	53 (18.93)			148 (26.96)			201 (24.25)		
II	95 (33.93)			230 (41.89)			325 (39.20)		
III	64 (22.86)			110 (20.04)			174 (20.99)		
IV	68 (24.29)			61 (11.11)			129 (15.56)		

*F* females, *M* males, *SD* standard deviation, *AFP* alpha fetoprotein, *TNM* tumor, nodes, metastasis-classification

^a^Kruskal-Wallis test for continuous variables

^b^Chi square test for categorical variables

The committee of ethics in Guangdong Medical University authorised the experimental and research protocols of this study. Experiments on humans were performed in accordance with relevant guidelines and regulations. After clearly explaining the purpose of the study to the participants, all controls and patients (or relatives of patients who already died) provided written informed consent. The study also adhered to tenets in the Helsinki declaration. All potential participants who declined to participate or ended up not participating were eligible for treatment, and non-participation did not result in any disadvantages for patients.

### Targeted next-generation sequencing (NGS) and identification of genetic variants

Aliquots of buffy coat and plasma separated from blood samples were stored at − 80 °C until subsequent treatment. All samples were included in the combined study. Genomic DNA was extracted from peripheral whole blood cells using the QIAamp system (QIAGEN Co.). Genomic DNA from 255 controls and 280 HCC patients were randomly sheared by sonication to an average size of 250 bp per fragment. Target enrichment technology was used as described by Anna Kiialainen et al. [16]. The enriched libraries were loaded onto the HiSeq system 2000 and approximately 90-bp paired-end reads were produced using the NGS technology (Illumina Genome Analyzer). We will use fastq short reads to align the NCBI build 37.1 hg19 [17]. Single-nucleotide variants (SNV) that obey the criteria that a. *P* for Hardy–Weinberg equilibrium (HWE, <10^− 4^), b. duplicated paired-end reads, c. overall depth ≤ 8×, d. SNP within 10 bp of a gap, or e. copy number variant ≥2 were then filtered [18]. For these concerns, only qualified SNPs were considered for this evaluation, so a 164-SNP set was used for the primary case-control study. Plink was used to calculate single-nucleotide variants [19], and the Haploview was used to perform visualisation [20].

### Population risk evaluation, linkage disequilibrium (LD) mapping and gene–gene interactions

We used the chi-square and Mann–Whitney U tests to compare and evaluate the clinical data between the patients and controls in discovery, replication and the combined groups. The risk evaluation was assessed using the Pearson chi-square test. Because 164 SNPs were genotyped, the Bonferroni-corrected *P* value for association studies is 0.05/164 = 0.0003 for single SNPs.

A gene–gene interaction in this study is defined as an SNP–SNP interaction and was conducted with LD mapping. To estimate the degree of LD between pairs of loci, the standardised disequilibrium coefficient (D′) was calculated and haplotype blocks were defined using the Haploview programme [20]. The haplotypic imputation, reconstruction and frequency estimations were conducted with an expectation–maximisation algorithm [21]. n_e_ = 1/∑_*P*i_^2^ was used to calculate the number of effective haplotypes, and Pi was the estimate of individual haplotype frequency [22]. Pi was calculated because the phase of the genotype was known and it was chosen in compliance with the homologous probabilities of occurrence that had a higher likelihood (>0.95 as cut-point).

### Permutation test and quantile–quantile (Q–Q) analysis

We performed permutation tests for 10^5^ permutations, in which subjects’ phenotypes were randomly realigned. *P* values (permutation or empirical *P* values) were specified as permutation values that were at least as extreme as the original statistics divided by the total permutation numbers. For better estimation of empirical *P* values, SNPs were reconsidered with 10^5^ permutations. Permutations were used to redistribute controls and patients. By convention if *P* < 0.05, the difference was considered statistically significant.

A Q–Q plot was then graphed to check the *P* value distribution. The ‘cumulative distribution function’ of the normal density and qth quantile of a Gauss distribution was signified by Φ(z) and ξ_q_, respectively, (Φ(ξ_q_) = q). Therefore, the probability <ξq is actually just q. The theoretical quantile was defined by the inverse of the normal cumulative distribution function. Especially, the theoretical fitting the empirical quantile z_(i)_ should be

$$ {\xi}_q=q\approx \frac{i-0.5}{n}, $$

$$ \mathrm{for}\;i=1,2,3,\dots, \mathrm{n}. $$

### SNP selection and TaqMan genotyping in the following replication study

SNPs in *CHD5* were selected on the basis of ‘significant SNPs’ found in the discovery-targeted NGS results of 255 controls and 280 HCC samples. Next, genomic DNAs from all other subjects (510 controls and 549 patients) were genotyped using TaqMan probes with the ABI 7500 Fast System (Applied Biosystems, forster City, CA) for the selected two SNPs in haplotypic block 3 (rs12564469 and rs9434711). PCRs were performed with 50 ng DNA in 25-ul total volume containing 0.25 ul Taq polymerase, 2.5 ul PCR mix, 0.625 ul of each primer and 2.5 ul dNTPs for 40 cycles of denaturation (95 °C) for 10 min, annealing (92 °C) for 15 s and extension (60 °C) for 1 min. Associations of the potential risk SNPs or haplotypes with HCC were further evaluated by stratification analysis with subgroups of age, sex, smoking and drinking status. Pi was defined as the division of the two P numbers, which means the larger in absolute terms indicating more meaningful value.

### False-positive report probability (FPRP) analysis

To avoid the possibility of false-positives inherent to performing multiple tests, a Bayesian statistical test-the FPRP-was performed for all significance in genetic association studies [23]. According to the method proposed, an FPRP value of ≤0.2 was regarded as pointing to a significant association, and a prior probability of 0.1 to check ORs of 1.50/0.67 was applied for risk/protective functions. The statistical power was calculated according to the case/control numbers and OR/*P* values in the study.

### Gene–environment interactions

The possible gene–environment interactions with high-order in the associations were evaluated using the multiple dimension reduction (MDR) programme [24]. Briefly, we carried out a 100-fold cross-validation and 1000-fold permutations under the assumption of no association. The maximum cross-validation consistency (CVC) and minimum average prediction error were requirements for the best interaction model.

### Statistical software

The SPSS 22.0 for Windows (SPSS, Chicago, IL) and R scripts (3.0.2 Suite) software were used for statistical analyses.

## Results

### Population association risk (PAR) in the discovery study

**Fig. 1:**
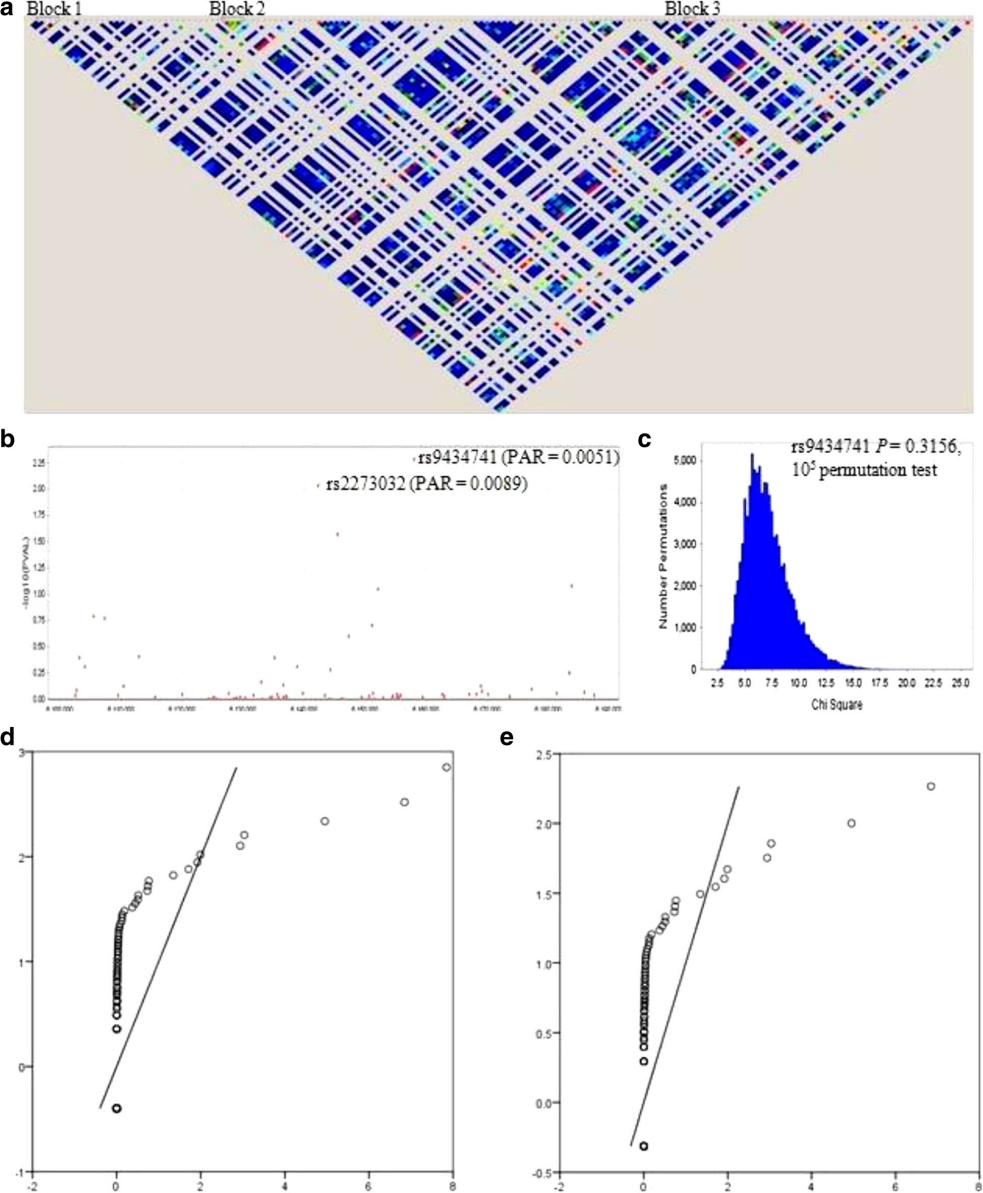
*CHD5* LD mapping and analysis in the discovery study. **a** LD mapping. **b** Manhattan plot. The –log_10_*P* values were for the association of each SNP with HCC, from two-sided Cochran–Armitage tests for trend. **c** 10^5^ permutation tests for the association analysis. **d** and **e** Q-Q plots for the test statistics of observed Chi-square values against expected Chi-square values (E, removing rs9434741)

We detected a total of 164 single-base substitutions analysing the targeted NGS results (Fig. 1a and Additional file 1: Table S1). Of these, eight were in a promoter region, 129 were intronic and 27 were in coding exons. A case-control study was conducted and the results indicated potential associations between the risk of HCC and the *CHD5* polymorphisms rs9434741 (PAR = 0.0051), rs2273032 (PAR = 0.0089) and rs12067480 (PAR = 0.0261) in the Han population (Fig. 1b and Additional file 1: Table S1). But they lost statistical significance after performing a Bonferroni correction. They also lost their significance after 10^5^ permutation tests (for example, *P* = 0.3156 for rs9434741, Fig. 1c). Q–Q plots were used to compare with the observed chi-square results with the distribution expected under the null hypothesis, there was deviation from expectation at a higher value of approximately 2.8 (Fig. 1d). After removing rs9434741, there were no significant curve changes compared with the expected distribution (Fig. 1e).

### LD and haplotypic analysis in the discovery study

Direct sequencing results revealed a total of 164 SNPs in *CHD5*. We identified three blocks with high LD (Fig. 1a). Block 1 includes SNP3–SNP6 (rs12037962, rs11587, rs41307753 and rs3810989). Block 2 includes SNP35–SNP38 (rs2273041, rs2273040, rs2273038 and rs55930553). Block 3 includes SNP115 and SNP116 (rs12564469 and rs9434711). Blocks were reconstructed according to their frequencies. The results of the haplotype-based case-control study between the HCC and control groups are shown in Table 2. We found that a haplotype AG in block 3 showed a significant association with HCC (*P* = 1.94 × 10^− 5^). It remained significant according to unconditional logistic regression analysis after adjustment for age, sex, smoking and drinking status (*P*_corrected_ = 5.73 × 10^− 5^) and after 10^5^ permutation tests (*P* = 4.00 × 10^− 5^).

**Table 2:** Haplotype frequencies in the discovery, replication and combined studies

Haplotypes	Case, Control Ratio Counts^a^	Case, Control Frequencies^b^	Chi Square	*PAR*	*P*_corrected_^c^	*P*_Permutation_^d^
Discovery study						
Block 1						
GGCA	176.0: 384.0, 171.1: 338.9	0.314, 0.335	0.54	0.4623	0.2970	0.9976
GACA	176.0: 384.0, 171.1: 338.9	0.314, 0.335	0.54	0.4623	0.2970	0.9976
AGCG	67.5: 492.5, 61.1: 448.9	0.121, 0.120	0.002	0.9651	0.8263	1
AACG	67.5: 492.5, 61.1: 448.9	0.121, 0.120	0.002	0.9651	0.8263	1
AGCA	29.5: 530.5, 20.9: 489.1	0.053, 0.041	0.795	0.3727	0.6037	0.9876
AACA	29.5: 530.5, 20.9: 489.1	0.053, 0.041	0.795	0.3727	0.6037	0.9876
Block 2						
CCCG	417.3: 126.7, 375.9: 118.1	0.767, 0.761	0.055	0.8153	0.7749	1
TTTA	42.0: 502.0, 37.3: 456.7	0.077, 0.076	0.011	0.9171	0.9690	1
CCTG	36.0: 508.0, 35.5: 458.5	0.066, 0.072	0.128	0.7208	0.8452	1
TTCA	25.5: 518.5, 25.2: 468.8	0.047, 0.051	0.098	0.7544	0.7805	1
CCCA	9.5: 534.5, 8.4: 485.6	0.017, 0.017	0.002	0.9622	0.9417	1
TTTG	7.7: 536.3, 5.8: 488.2	0.014, 0.012	0.121	0.7282	0.7548	1
Block 3						
AA	324.0: 170.0, 289.0: 151.0	0.656, 0.657	0.001	0.9757	0.8983	1
GG	143.8: 350.2, 148.8: 291.2	0.291, 0.338	2.399	0.1214	0.1665	0.5747
AG	26.2: 467.8, 2.2: 437.8	0.053, 0.005	18.248	1.94 × 10^− 5^	5.73 × 10^−5^	4.00 × 10^−5^
Replication study						
Block 3						
AA	630.9: 341.1, 579.0: 309.0	0.649, 0.652	0.018	0.8945	0.8714 ^8^	0.9893
GG	294.5: 677.5, 303.5: 584.5	0.303, 0.342	3.202	0.0735	0.1069	0.1542
AG	46.6: 925.4, 5.5: 882.5	0.048, 0.006	29.716	5.038 × 10–^8^	7.571 × 10-	0.00001
Combined study						
Block 3						
AA	954.9: 511.1, 868.0: 460.0	0.651, 0.654	0.015	0.9012	0.9467	0.9909
GG	438.3: 1027.7, 452.3:875.7	0.299, 0.341	5.556	0.0184	0.0383	0.0410
AG	72.7: 1393.3, 7.7: 1320.3	0.050, 0.006	47.941	4.393 × 10^−12^	5.514 × 10^− 11^	0.00001

Block 1, rs12037962, rs11587, rs41307753 and rs3810989

Block 2, rs2273041, rs2273040, rs2273038 and rs55930553

Block 3, rs12564469 and rs9434711

^a^Number of haplotypes were compared in cases versus controls: Haplotype(1):haplotype(others) cases, Haplotype(1):haplotype(others) controls

^b^Frequency of the haplotype

^c^Calculated in logistical regression models with adjustment for age, gender, smoking and drinking status; *p* < 0.005 means significant value by Bonferroni correction based on the total number of markers genotyped

^d^Empirical *p*-value based on 10^5^ permutations of case-control status using the max(T) procedure. *p* < 0.05 means significant value

### Population association and haplotypic analysis based on selected SNPs in the replication and combined studies

We selected SNPs rs12564469 and rs9434711 in block 3 from the first SNP discovery study for the next study. Replicative results showed no associations for rs12564469 (PAR = 0.0800, *P*_adjusted_ = 0.1029, *P*_Permutation_ = 0.1062) or for rs9434711 (PAR = 0.8718, *P*_adjusted_ = 0.8485, *P*_Permutation_ = 0.9601). Finally, a combined study including discovery and replicative cohort data was conducted. Combined results also showed no association for rs12564469 (PAR = 0.0210, *P*_adjusted_ = 0.0290, *P*_Permutation_ = 0.0286) and for rs9434711 (PAR = 0.8829, *P*_adjusted_ = 0.9137, *P*_Permutation_ = 0.9704; Table 3).

**Table 3:** rs12564469 and rs9434711 in replication and combined studies

	Alleles^a^	Case, Control Ratio Counts^b^	Case, Control Frequencies^c^	*Chi square*	PAR^d^	*P*_adjusted_^e^	*P*_permutation_^f^
Replication							
rs12564469	A > G	659:289, 570:298	0.695, 0.657	3.065	0.0800	0.1029	0.1062
rs9434711	A > G	341:629, 309:579	0.352, 0.348	0.026	0.8718	0.8485	0.9601
Combined							
rs12564469	A > G	1003:431, 857:445	0.699, 0.658	5.328	0.0210	0.0290	0.0286
rs9434711	A > G	511:953, 460:868	0.349, 0.346	0.022	0.8829	0.9137	0.9704

^a^The major allele is listed first, then the minor allele

^b^Number of alleles were compared in cases versus controls: allele(1):allele(2) cases, allele(1):allele(2) controls

^c^Frequency of the association allele

^d^PAR, population attributable risk

^e^Calculated in logistical regression models with adjustment for age, gender, smoking and drinking status

^f^*P* for 10^5^ permutation test

The results of the haplotype-based replication and combined studies between the HCC and control groups are shown in Table 2. We observed increased frequencies of haplotype AG in HCC patients compared with those seen in healthy controls both in the replication study (PAR = 5.038 × 10^− 8^, *P*_adjusted_ = 7.571 × 10^− 8^, *P*_Permutation_ = 0.00001) and in the combined study (PAR = 4.393 × 10^− 12^, *P*_adjusted_ = 5.514 × 10^− 11^, *P*_Permutation_ = 0.00001).

### Stratification analysis of haplotypes

The association of haplotype AG (block 3) with the risk of HCC in subgroups such as age, sex, smokers and drinkers were evaluated further using replication and combined studies (Table 4). We found that those individuals carrying haplotype AG had a significantly increased risk of HCC, and the risk was increased in patients of >55 years (*P* = 6.04 × 10^− 8^ and *P*_i (P2/P1)_ = 5.12 × 10^− 4^) and in drinkers (*P* = 9.43 × 10^− 8^ and *P*_i (P2/P1)_ = 3.25 × 10^− 6^).

**Table 4:** Stratification analysis for associations between block 3 (rs12564469-rs9434711) haplotypes and HCC risk in the discovery, replication and combined studies

Variables	Discovery study				Replication study				Combined study			
	Cases/controls		OR (95% CI)	*P*	Cases/controls		OR (95% CI)	*P*	Cases/controls		OR (95% CI)	*P*
	AG	AA+GG			AG	AA+GG			AG	AA+GG		
Age (ys)												
≤55	10/0.6	123.2/128.8	8.57 (1.28–76.03)	0.010	13.2/1.7	231.5/198.5	5.11 (1.20–23.49)	0.026	23.2/2.3	354.7/327.3	9.62 (2.37–41.84)	1.18 × 10^− 4^
>55	16.2/1.6	344.6/309	6.39 (1.60–28.85)	0.007	33.4/3.8	651/684	7.88 (2.64–20.18)	8.23 × 10^− 5^	49.6/5.4	995.6/993	9.13 (3.36–22.70)	6.04 × 10^− 8^
*Pi*				0.7				3.17 × 10^−3^				5.12 × 10^−4^
Gender												
Females	6/0.2	70.2/139.6	2.81 (2.22–3.47)	0.008	13.6/1.8	231.4/268	7.35 (1.76–33.38)	0.008	19.6/2	301.6/407.6	12.43 (3.03–54.68)	6.36 × 10^−5^
Males	20.2/2	397.6/298.2	7.15 (1.55–29.07)	0.009	33/3.7	694/614.5	7.09 (2.38–17.14)	7.95 × 10^−4^	53.2/5.7	1091.6/912.7	7.35 (3.02–15.98)	8.61 × 10^−7^
*P*i				1.125				0.099				0.014
Drinking												
Never	3/2.2	320.2/333.8	1.78 (0.30–11.79)	0.505	9.6/1.5	574.4/652	5.17 (1.19–22.93)	0.025	12.6/3.7	894.6/985.8	3.36 (1.11–9.83)	0.029
Ever+current	23.2/0	147.6/104	1.64 (1.47–1.75)	1.07 × 10–4	37/4	351/230.5	5.88 (2.03–16.01)	8.76 × 10^−5^	60.2/4	498.6/334.5	9.78 (3.56–24.83)	9.43 × 10^−8^
*P*i				2.12 × 10^−4^				3.50 × 10^−3^				3.25 × 10^−6^

*P, P* value for haplotype model, which obtained in logistic regression with adjustment for age, sex, smoking status and drinking status

*Pi, means P*_*2*_ */P*_*1*_

### FPRP

The significant associations of FPRP values for block 3 haplotype AG (vs. AA + GG) at different levels of prior probability are listed in Table 5. FPRP values of haplotype AG for HCC risk in patients >55 years were <0.20 for the assigned prior probability (0.017 for the prior probability of 0.1 in the replication study; 0.004 and 0.010 for the prior probabilities of 0.1 and 0.01, respectively, in the combined study). For the risk of HCC in alcohol drinkers, when the assumptions of prior probability were 0.1 and 0.01, all findings were significant not only in the discovery study but also in the replication and combined studies (FPRP < 0.20). Moreover, when the assumption of prior probability was 0.001, this association was still prominent in the combined study (FPRP = 0.069).

**Table 5:** FPRP values for associations between HCC risk and block 3 haplotype frequencies (AG vs. AA+GG)

Variables	Statistical power^a^	Prior probability			
		0.1	0.01	0.001	0.0001
HCC risk in >55 years old group					
Discovery study	0.704	0.216	0.493	0.721	0.885
Replication study	0.689	0.017	0.271	0.525	0.843
Combined study	0.837	0.004	0.010	0.347	0.706
HCC risk in drinking group					
Discovery study	0.792	0.003	0.013	0.298	0.635
Replication study	0.658	0.005	0.017	0.424	0.757
Combined study	1	< 0.001	0.005	0.069	0.236

Block 3, rs12564469 and rs9434711

If the prior probability <0.20, the results in FPRP are in bold

### Association of high-order interactions with HCC risk by MDR

The interactions of high-order assessed with MDR were conducted, including the potential risk haplotype AG and four known risk factors (age, sex, smoking and drinking status), in order to check whether possible gene–environmental interactions in association with the risk of HCC exists. In the discovery study, we noticed that the best one-factor model was drinking status, with the highest CVC (99/100, the same model is selected as the best model 99 out of 100 times) and the lowest prediction error (0.385). The best model for two-factors was drinking status plus haplotype AG, with the highest CVC (96/100) and the lowest prediction error (0.403). Interestingly, the model with 5-factors had a maximum CVC (100/100) and a minimum prediction error (0.378). This is a model with better prediction than the model with one factor. Same results were found in the replication study and the combined study (Table 6).

**Table 6:** MDR analysis for the prediction of HCC risk with and without haplotype AG

Best interaction models	Cross-validation	Average prediction error	*P* ^a^
Distcovery study			
1	99/100	0.385	< 0.0001
1,2	96/100	0.403	< 0.0001
1,2,3	100/100	0.401	< 0.0001
1,2,3,4	87/100	0.380	< 0.0001
1,2,3,4,5^b^	100/100	0.378	< 0.0001
Replication study			
1	95/100	0.412	< 0.0001
1,2	94/100	0.417	< 0.0001
1,2,3	98/100	0.389	< 0.0001
1,2,3,4	90/100	0.383	< 0.0001
1,2,3,4,5^b^	100/100	0.368	< 0.0001
Combined study			
1	96/100	0.399	< 0.0001
1,2	94/100	0.410	< 0.0001
1,2,3	99/100	0.396	< 0.0001
1,2,3,4	89/100	0.382	< 0.0001
1,2,3,4,5^b^	100/100	0.375	< 0.0001

Labels: 1, drinking status; 2, haplotype AG (block 3); 3, age; 4, smoking status; 5, gender

^a^*P* value for 1000-fold permutation test

^b^The best model with maximum cross-validation consistency and minimum prediction error rate

## Discussion

Studies have found that the chromosome aberration of 1p36 deletion is not frequent in HCC. It remains to be determined whether the common SNPs in *CHD5* are associated with the risk of HCC. *CHD5* is a tumour-suppressing gene of the chromodomain gene family, first identified as a tumour-suppressing gene mapping to 1p36.31 [25].

The integration of clinical phenotypes and genomic information may enable precision cancer medicine through NGS approaches [26]. Results of our targeted NGS and TaqMan genotyping revealed no significant associations with the risk of HCC neither in the discovery study nor in the replication and combined studies. For two data sets, it is important to identify whether the hypothesis of a common distribution is proven to be true. The Q–Q plot offers more insight into the discrepancy than any other statistical analysis such as the Kolmogorov Smirnov 2-sample test or the chi-square test. However, we did not find any significant change after removing rs9434741, which suggests that the most likely associated SNP is not a risk locus.

Nonetheless, we inadvertently found a positive association of a rare haplotype AG (block 3: rs-12564469-rs9434711) in *CHD5* and HCC, which has not been reported to date. Importantly, this association quality increased in a gene dose-dependent manner as the number of samples increased (*PAR* in Table 2). Thus, our results support the idea that the 1p36 region plays a role in HCC. We believe it is possible that hereditary mutations of tumour-suppressing genes in the 1p36 region contribute to the aggressive properties of liver cancer. Hereditary changes in the 1p36 region are extraordinarily common in human tumours, occurring in malignancies of epithelial, neural and haematopoietic origin [25]. Genetic mutations of the tumour-suppressing gene *CHD5* have conduced to the understanding of human oncogenesis.

It seems that the risk effect of the haplotype AG was more evident in the drinkers’ subgroup (Ref: *P*_i_ in Table 4) with the stratified analysis. One of the possible comments is that the sample size is smaller in subgroups. Nevertheless, the results of the FPRP analysis for those findings showed that the drinkers group remained significant at the prior probability level of 0.1. We believe that in drinkers, alcohol-related carcinogens may cause DNA damage [27] and that accumulated DNA damage caused by the regular carcinogenic exposure to alcoholic drinks [28, 29] might enhance the effect of genetic instability.

Next, we conducted a high-order gene (haplotype)–environment interaction analysis with MDR testing to support the above results. The best interaction model revealed that the *CHD5* haplotype AG interacted with the drinking status with a maximal CVC and minimal prediction error, which was more obvious in the interaction entropy analysis. Our results suggested that the stratification testing reliably identified alcohol drinking as a risk factor.

Our recent study had reported that the *CHD5* rs12564469-rs9434711 region might functionally contribute to HCC prognosis and *CHD5* mRNA expressions [30]. It is possible that *CHD5* plays an essential role in cancer development. The expression of multiple genes that regulate pathways in the tumourigenic process was modulated by CHD5 [31]. Apoptosis, cellular senescence and neonatal death will occur by excessive activation of these tumour-suppressive pathways, dependent on p53, p19 and p16. CHD5 expression seems to be restricted to neural-derived tissues, as opposed to CHD4 which is expressed in all tissues. CHD5 mRNA cannot be detected in the liver, placenta, spleen, bone marrow, thyroid, stomach, pancreas, small intestine, colon or prostate [8, 30]. Because of this, expression of the candidate tumour-suppressing genes was sequentially disrupted by specific shRNAs. What is more, CHD5 expression is down-regulated in HCC tissues and HepG2, and the expression level of CHD5 was inversely correlated with the expression of oncogene miR-454 in HCC tissues [32]. Therefore, CHD5 as the cause of the observed phenotype was identified.

Alternatively, CHD5 or a CHD5-containing complex could interact with p53 directly. A similar model for a MTA2-containing NuRD complex regulating the p53-mediated transactivation by modulating the p53 acetylation status [33] was suggested. CHD5 may function in a similar manner since it was shown to be part of a NuRD-like complex [34]. Both the interactions and functions are equally important for the development of HCC. The genetic engineering mice with a heterozygous deficiency of the (human) 1p36 locus were prone to develop non-neural tumours (lymphoma, squamous cell carcinoma and hibernoma). *CHD5* was found to positively regulate p53 presumably via p14/p19ARF [35, 36]. But the exact molecular mechanisms could not be defined.

## Conclusions

In short, we identified a rare haplotype in *CHD5* that was significant associated with the risk of HCC. Our results highlight the breadth of precision medicine by providing clues to help the advancement of effective diagnostic, management and prevention tools against cancer. Nonetheless, larger sample size studies are needed to corroborate our findings.

## Additional file

Additional file 1: **Table S1.** A case-control study in the discovery study with targeted NGS. (XLS 37 kb)

## Abbreviations

*CHD5*: *Chromodomain helicase DNA-binding protein 5*
FPRP: False-positive report probability Population risk evaluation
HCC: Hepatocellular carcinoma
LD: Linkage disequilibrium
MDR: Multiple dimension reduction
NGS: Targeted next-generation sequencing
Q-Q: Permutation test and quantile-quantile
SNPs: Single-nucleotide polymorphisms
